# Poststroke Hip Fracture: Prevalence, Clinical Characteristics, Mineral-Bone Metabolism, Outcomes, and Gaps in Prevention

**DOI:** 10.1155/2013/641943

**Published:** 2013-09-25

**Authors:** Alexander Fisher, Wichat Srikusalanukul, Michael Davis, Paul Smith

**Affiliations:** ^1^Department of Geriatric Medicine, The Canberra Hospital, P.O. Box 11, Canberra, Woden, ACT 2606, Australia; ^2^Australian National University Medical School, P.O. Box 11, Canberra, Woden, ACT 2606, Australia; ^3^Department of Orthopaedic Surgery, The Canberra Hospital, P.O. Box 11, Canberra, Woden, ACT 2606, Australia

## Abstract

*Objective*. To assess the prevalence, clinical and laboratory characteristics, and short-term outcomes of poststroke hip fracture (HF). *Methods*. A cross-sectional study of 761 consecutive patients aged ≥60 years (82.3 ± 8.8 years; 75% females) with osteoporotic HF. *Results*. The prevalence of poststroke HF was 13.1% occurring on average 2.4 years after the stroke. The poststroke group compared to the rest of the cohort had a higher proportion of women, subjects with dementia, history of TIA, hypertension, coronary artery disease, secondary hyperparathyroidism, higher serum vitamin B12 levels (>350 pmol/L), walking aid users, and living in residential care facilities. The majority of poststroke HF patients had vitamin D insufficiency (68%) and excess bone resorption (90%). This group had a 3-fold higher incidence of postoperative myocardial injury and need for institutionalisation. In multivariate analysis, independent indicators of poststroke HF were female sex (OR 3.6), history of TIA (OR 5.2), dementia (OR 4.1), hypertension (OR 3.2), use of walking aid (OR 2.5), and higher vitamin B12 level (OR 2.3). Only 15% of poststroke patients received antiosteoporotic therapy prior to HF. *Conclusions*. Approximately one in seven HFs occurs in older stroke survivors and are associated with poorer outcomes. Early implementation of fracture prevention strategies is needed.

## 1. Introduction

Stroke and osteoporotic hip fracture (HF) are two highly prevalent conditions among the elderly, both with poor clinical outcomes and high medical and social costs. Worldwide the prevalence of these events is expected to increase dramatically because of rapid population ageing. Osteoporosis and low-impact trauma are the main determinants of HF in the elderly. Stroke increases the risk of falls [1–5] as a result of impaired locomotor function and muscle weakness [6, 7], accelerates bone loss (especially in the hemiplegic leg) [8–26], and subsequently leads to fractures [4, 27–29]. Although accumulating evidence suggests bidirectional links between vascular diseases, including stroke, and osteoporotic fractures [9–11, 30–35], there is still considerable uncertainty regarding the contribution of stroke to osteoporotic HF.

The incidence of HF after a stroke was reported to be 1.5–4 times [27, 29, 36, 37] or even >7 times [28] higher than in the general population, but this was not observed in other studies [38–40]. Similarly, the reported prevalence of previous stroke among patients with HF ranges from 3% to 38.5% [4, 41–43].

Data concerning clinical characteristics, outcomes, prediction, and prevention of poststroke HF are also inconsistent [3, 35, 41, 44, 45]. Most previous studies have examined only a small number of the many clinical and pathogenic factors involved in stroke and HF. The exact aetiology and pathogenesis of poststroke osteoporosis remain not fully understood. Studies have generally focused on bone mineral density (BMD) and documented accelerated bone loss, especially in paretic limbs [10, 12–14, 22], with only few analysing mineral and bone metabolism in poststroke patients [15, 21, 46, 47]. Fracture prevention and osteoporosis care are not included in current guidelines for stroke management.

The aims of this study were (1) to investigate the prevalence of poststroke HF, (2) to compare in HF patients with and without a previous stroke the demographic, clinical, and laboratory characteristics and short-term outcomes, (3) to test in both groups the associations of parameters related to mineral and bone metabolism, and (4) to determine whether any clinical or laboratory characteristics are indicative/predictive for a poststroke HF. 

## 2. Materials and Methods

### 2.1. Study Patients

A total of 847 consecutive patients aged 60 years and older who were admitted to the Canberra Hospital with low-trauma HF and underwent surgery were investigated. After the exclusion of 86 patients with pathological HF (metastatic bone cancer, multiple myeloma, and Paget's disease) or primary hyperparathyroidism, a total of 761 patients (570 women and 191 men) were included in the analysis. All patients or their guardians gave informed consent to undergo examination and surgical treatment. The study was conducted according to the Helsinki Declaration and approved by the regional Human Research Ethics Committee. 

### 2.2. Clinical Data Collection

The data were collected prospectively: demographic, medical, lifestyle, and residential characteristics, and in-hospital management, peri-operative complications and short-term outcomes were recorded. In all patients a detailed medical history and full physical examination were performed. The presence of comorbidities was identified based on clinical manifestations, review of hospital medical records, general physician's progress notes/letters, and medication used. Data on a history of previous stroke and transient ischaemic attack (TIA) were initially abstracted from the medical records. A patient was included in the group with a history of stroke if he met the following criteria: (1) presence of neurological deficit consistent with a stroke and/or (2) medical documentation confirming a stroke before the HF. 

Short-term outcome measures included (1) postoperative myocardial injury as defined by cardiac troponin I rise (cTnI > 0.06 *μ*g/L), (2) prolonged length of stay (LOS ≥ 20 days), (3) discharge to a permanent residential care facility (RCF) for patients who were admitted from home, and (4) all-cause in-hospital death.

### 2.3. Sample Collection and Methods of Laboratory Analyses

In each patient venous blood and second morning urine samples were collected under standardised conditions after a 12-hour overnight fast usually within 24 hours after arrival at the emergency department. After centrifugation of blood, one serum sample as well as the urine sample was frozen and stored at −70°C until further analysis. Routine haematological and biochemical assessments were performed by standardized methods on autoanalyzers at the day of collection. 

All patients had the following tests performed: complete blood count, urea, creatinine and electrolytes, liver function tests, fasting blood glucose (and HbA_1C_ in diabetic patients), thyroid function tests (TSH, T_4_, and T_3_ if indicated), C-reactive protein (CRP), and cTnI (two-step chemiluminescent microparticle immunoassay, Chemiflex, Abbott Labs, Mississauga, Ontario, Canada). Glomerular filtration rate (eGFR) was calculated using a standardized serum creatinine-based formula normalized to a body surface area of 1.73 m² [48]. Full blood count, urea, creatinine and electrolytes, serum cTnI, and CRP were also assessed within 24 hours postoperatively and then after if clinically indicated.

In order to evaluate mineral and bone metabolism in all patients, the following biochemical parameters were determined: serum concentrations of 25(OH) vitamin D (25(OH)D), intact PTH, total calcium, phosphate, magnesium, osteocalcin (OC), and bone-specific alkaline phosphatise (BAP) as markers of bone formation and urinary concentrations of deoxypyridinoline (DPD/Cr), and cross-linked N-telopeptide of type 1 collagen (NTx/Cr) as markers of bone resorption (both corrected for urinary creatinine concentrations in the same sample). Serum 25(OH)D was measured by radioimmunoassay kit (Dia Sorin, Stillwater, MN, USA; sensitivity 0.7 nmol/L) and intact PTH by two-site chemiluminescent enzyme-linked immunoassay on DPC Immulite 2000 (Diagnostic Products Corp, Los Angeles, CA, USA; sensitivity 0.07 pmol/L). Serum OC was determined by electrochemiluminescent immunoassay (Elecsys 1010, Roche Diagnostics, Ltd. Corp., IN, USA), serum BAP by Metra BAP ELISA (Quidel Corp., San Diego, CA, USA), urinary NTx by ELISA (Wampole Labs, Princeton, NJ, USA), and urinary DPD by 2-site chemiluminescent enzyme-labelled immunoassay (DPC Immulite 2000, Diagnostic Products, Los Angeles, CA, USA). All samples were analysed with commercially available kits of the same lot number according to the manufacturer's protocol and blind to any clinical information. In these methods both the intra- and interassay coefficient of variations (CVs) ranged from 2.1% to 12.7%. Serum calcium concentrations were corrected for serum albumin. 

For the analyses, insufficiency of vitamin D was defined as 25(OH)D < 50 nmol/L and deficiency as 25(OH)D < 25 nmol/L based on current recommendations. Secondary hyperparathyroidism (SHPT) was defined as elevated serum PTH (>6.8 pmol/L, the upper limit of the laboratory reference range). For levels of bone turnover markers, we used the standard laboratory reference ranges and data provided by the manufacturer. In the analyses chronic kidney disease (CKD) was defined as eGFR < 60 mL/min/1.73 m² (CKD stage ≥ 3). To express the balance between bone formation and resorption, the data of biochemical bone turnover markers were evaluated as *Z*-scores, and a bone balance status index (BSI) was calculated [49, 50]. The formula for *Z*-scores was *Z* = (*x*_*i*_ − *X*)/SD, where, *x*_*i*_ was the patient's value of a marker and *X* and SD were the normal mean and standard deviations for this parameter, respectively. For BSI the formula was BSI = (*Z*-scores  of  OC + BAP)/2 − (*Z*-scores  of  DPD/Cr + NTx/Cr)/2.

### 2.4. Statistical Analyses

Descriptive statistics included means and standard deviations (SD) for continuous variables or percentages for categorical variables. Comparisons between groups were performed using analysis of variance and Student's *t-test* (for continuous variables) and *χ* ^2^ test (for categorical variables). Pearson's correlation coefficient with log-transformed data (to achieve normal distribution) was used to study the correlation between variables; Bonferroni and Sidak adjustments for multiplicity have been performed. Univariate and multivariate linear regression analyses were performed to determine the associations between different parameters related to mineral and bone metabolism in patients with and without history of stroke; all potential confounding variables (demographic, clinical, and biochemical) with statistical significance ≤0.20 on univariate analyses were included in multivariate analysis. The relationships between presence of poststoke HF and clinical characteristics as well as different laboratory parameters as independent variables were investigated by linear regression analyses. To quantify the significance of multicollinearity phenomena in regression analysis, the variance inflation factor was calculated. Two-sided *P* < 0.05 values were considered statistically significant. The Stata software version 10 (StataCorp, College Station, TX, USA) was used for all statistical analyses.

## 3. Results

### 3.1. Prevalence and General Clinical Characteristics of Patients with Poststroke Hip Fracture

Of 761 older HF patients, 100 (13.1%) had a history of stroke, of whom 83.0% were women. The clinical characteristics of the patients according to stroke history are summarized in Table 1. HF occurred on average 2.4 ± 2.6 years after the stroke, in 68.0% on the hemiplegic side. Among poststroke HF patients, the proportion of females and individuals admitted from long-term residential care facilities (RCF) was significantly higher compared to the rest of the cohort. The predominance of women was not age related, as the age of women and men with poststroke HF did not differ, whereas in the nonstroke group females were significantly older (+2.1 year, *P* = 0.002). As would be expected, the group of poststroke HF patients has a higher prevalence of previous TIAs, dementia, hypertension, coronary artery disease (CAD), and myocardial infarction, American Society of Anaesthesiologists score ≥3 on admission, and using a walking device; atrial fibrillation was also more often among the poststroke patients, but the difference did not reach statistical significance. History of stroke was inversely related with male gender (Pearson correlation coefficient *r* = −0.151; *P* = 0.011) and positively associated with hypertension (*r* = 0.186, *P* = 0.002) and CAD (*r* = 0.128, *P* = 0.033). The two groups did not differ with regard to mean age, HF type, prevalence of diabetes mellitus, chronic obstructive pulmonary disease, Parkinson's disease, chronic kidney disease, alcohol consumption, and smoking habits.

Among 24 haematological and biochemical variables, including parameters of iron metabolism, vitamin B12, folic acid, liver, renal and thyroid functions, only mean values of leukocyte count and serum concentration of vitamin B12 were higher, and the mean corpuscular volume was lower in poststroke HF patients compared to the rest of the cohort (Table 2). 

The prevalence of anaemia (haemoglobin < 120 g/L: 32.0% versus 36.7%, *P* = 0.456), renal impairment (eGFR < 60 mL/min/1.73 m^2^: 47.0% versus 4.3.4%, *P* = 0.650), hypoalbuminemia (<33 g/L: 32.0% versus 27.9%, *P* = 0.637), low total lymphocyte count (<12 × 10^9^: 40.0% versus 44.7%, *P* = 0.548), low transferrin (<1.5 g/L: 33.0% versus 25.9%, *P* = 0.356), and low vitamin B12 levels (<250 pmol/L: 33.0% versus 40.8%, *P* = 0.395) was similar in both groups. In other wards, about one-third of HF patients with or without history of stroke were anaemic and undernourished, and nearly half have renal impairment (CKD stage ≥ 3).

On the other hand, the proportion of subjects with higher vitamin B12 levels (>350 pmol/L) was significantly higher among the poststroke HF patients (60.0% versus 39.4%, *P* = 0.032), although none of the patients had solid or haematological malignancies, serious liver diseases, or received parenteral nutrition, which might have been associated with elevated vitamin B12 serum levels [51–53].

### 3.2. Mineral and Bone Metabolism in Poststroke Hip Fracture Patients

History of stroke was associated with higher levels of serum PTH (Pearson correlation coefficient *r* = 0.155, *P* = 0.010), vitamin B12 (*r* = 0.139, *P* = 0.027), urinary bone resorption marker DPD/Cr (*r* = 0.183, *P* = 0.004), and lower BSI (*r* = −0.170, *P* = 0.009). In the poststroke group compared to the rest of the cohort (Table 3), the mean serum PTH levels (+34.3%) and the incidence of SPTH (+15.8%) were significantly higher, although the mean serum 25(OH)D concentrations were similar, and the incidence of vitamin D insufficiency (<50 nmol/L) was even slightly lower (−13.7%, *P* = 0.044). Interestingly, among the poststroke patients 56.0% of those with 25(OH)D < 50 nmol/L had SHPT, whereas in the nonstroke group elevated PTH was observed only in 36.0% of subjects with vitamin D insufficiency (*P* = 0.007, *χ* ^2^ test), indicating that in the former group in addition to low vitamin D levels other mechanisms contribute to the excess of PTH. In an age- and sex-adjusted model, serum PTH levels were significantly associated with history of stroke (OR = 1.058, 95%CI 1.00–1.11, *P* = 0.025) indicating that risk for poststroke HF relative to 1 pmol/L increase in PTH increases by 5.8%. Of note, the proportion of subjects with SHPT among HF patients without CVD was 23.2%. The odds ratio (adjusted for sex, age, dementia, 25(OH)D < 50 nmol/L, and DPD/Cr > 7.5 nmol/*μ*mol) for SHPT in the poststroke HF subjects compared to the later group was 4.34 (95%CI 1.63–11.52, *P* = 0.003). 

The mean serum concentrations of calcium (albumin corrected), phosphate, and magnesium were within normal ranges and similar in both groups. Regarding bone turnover status, compared to the rest of the cohort, patients with poststroke HF demonstrated a lower mean serum level of osteocalcin and higher mean levels of urinary bone resorption markers DPD/Cr (*P* = 0.004) and NTx/Cr (*P* = 0.056) resulting in a significantly higher negative BSI (resorption exceeds formation). The mean serum OC/BAP ratio, an indicator of osteoblastic differentiation [54], in the poststroke HF patients was also significantly lower. DPD/Cr was elevated (>7.5 nmol/*μ*mol) in 90.0.0% of poststroke patients, in 81.9% of the total nonstroke group and in 74.8% of the non-CVD group. NTx/Cr was elevated (>65 nmol/*μ*mol) in 78.0%, 71.6%, and 70.4%, respectively; osteocalcin was below the low limit of normal range (<14 ng/mL) in 57.0%, 51.9%, and 50.1%, respectively. In multiple regression analyses adjusted for age, sex, HF type, 25(OH)D, PTH, eGFR, calcium, phosphate, and magnesium, history of stroke was an independent determinant of both higher DPD/Cr levels (*β* = 3.87, 95%CI 1.11–6.64, *P* = 0.006) and lower BSI (*β* = −4.17, 95%CI −7.29–1.06, *P* = 0.027).

In the total cohort, higher B12 levels (>350 pmol/L) were associated with significantly lower mean serum OC/BAP ratio (0.63 ± 0.45 versus 0.78 ± 0.65, *P* = 0.036) and serum albumin concentrations (36.0 ± 6.5 versus 38.2 ± 5.7 g/L, *P* = 0.003). Poststroke patients with higher vitamin B12 levels had significantly higher BAP levels (28.4 ± 10.9 versus 21.0 ± 6.0 IU/L, *P* = 0.028). Compared to nonstroke subjects, the poststroke group with higher vitamin B12 levels had a lower OC/BAP ratio (0.54 ± 0.36 versus 0.65 ± 0.47, *P* = 0.025). 

### 3.3. Metabolic Relationships in Hip Fracture Patients with and without History of Stroke

The relationships between parameters of mineral and bone metabolism in HF patients with and without history of stroke were further analysed by Pearson correlation coefficients, and the results are displayed in Table 4. These demonstrate similarities as well as remarkable differences between the groups. In both groups, there was a significant negative correlation between log-transformed serum PTH levels and calcium, calcium correlated positively with osteocalcin, serum phosphate levels were associated positively with urinary DPD/Cr and negatively with eGFR, BAP correlated with vitamin B12, osteocalcin correlated negatively with eGFR, which inversely correlated with phosphate and age, the two urinary resorption markers were significantly interrelated, and both strongly correlated to BSI. However, only in poststroke HF patients, serum phosphate levels correlated positively with serum calcium, osteocalcin, and urinary NTx/Cr and negatively with BSI, and PTH correlated with vitamin B12 levels. No association was observed between 25(OH)D concentrations and any of bone turnover markers and BSI in the poststroke group, while in the nonstroke group 25(OH)D inversely correlated with each of the four bone markers and positively with BSI (all *P* < 0.03, not shown).

### 3.4. Clinical and Laboratory Variables Independently Associated with History of Stroke in Older Hip Fracture Patients

In multiple regression models, poststroke HF was designated as the dependent variable and all clinical, and laboratory characteristics with *P* ≤ 0.20 on univariate analysis were designed as the independent variables (Table 5). A multivariate logistic regression model which included only clinical characteristics revealed that the six following variables were independent indicators for history of stroke in HF patients: female sex, history of TIA, dementia, hypertension, CAD, and use of a walking aid. The mean variance inflation factor (VIF) of 1.19 indicates that multicollinearity was not significant in this model.

When the laboratory parameters were analysed as continuous variables and adjusted for age and sex, the significant independent associations with poststroke HF showed only vitamin B12 (*P* = 0.006), DPD/Cr (*P* = 0.035), and female sex (*P* = 0.011). In a similar model with laboratory parameters as categorical variables corrected for age and sex, higher vitamin B12 levels and female sex were the only significant independent indicators of history of stroke (Table 5, Model 2). The other variables identified in the univariate analysis dropped out of the model, presumably because of their strong correlations with these two factors.

In multiple regression analysis using both clinical and categorical laboratory characteristics as independent variables, all clinical factors as well as higher serum vitamin B12 levels retained substantial independent value for poststroke HF, except CAD which becomes borderline significant (*P* = 0.057). Of note, relation of higher serum vitamin B12 to poststroke HF in the final model adjusted for both clinical and laboratory confounding variables did not change. This suggests that higher levels of B12, in contrast to vitamin D insufficiency, SHPT, and excess bone resorption, contain information not captured in and complementary to clinical characteristics. The mean variance inflation factor (VIF) of 1.03 in the final model indicates that multicollinearity was not significant. 

### 3.5. Predicting Poststroke Hip Fracture

**Figure 1:**
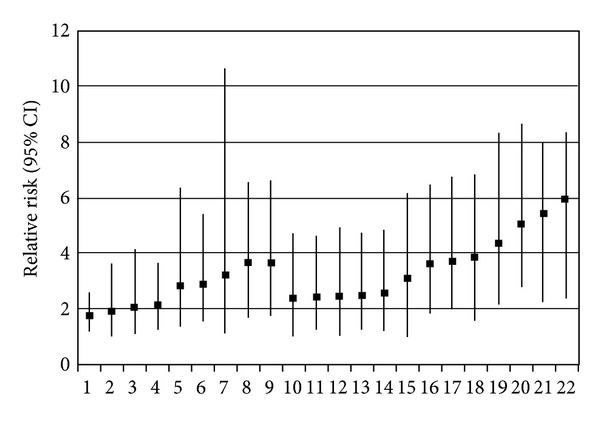
Estimated relative risk and 95% confidence intervals (CI) for poststroke hip fracture in older persons by selected clinical and laboratory characteristics. 1—Use of a walking aid; 2—coronary artery disease (CAD); 3—serum vitamin B12 > 350 pmol/L; 4—elevated serum PTH level (<6.8 pmol/L); 5—hypertension; 6—dementia; 7—female sex; 8—resident of a long-term care facility; 9—history of transient ischaemic attack; 10—CAD and serum vitamin B12 > 350 pmol/L; 11—hypertension and serum vitamin B12 > 350 pmol/L; 12—CAD and use of a walking aid; 13—CAD and hypertension; 14—use of a walking aid and elevated serum PTH level (<6.8 pmol/L); 15—transient ischaemic attack and elevated serum PTH level (<6.8 pmol/L); 16—dementia and use of a walking aid; 17—dementia and female sex; 18—transient ischaemic attack and hypertension; 19—dementia and serum vitamin B12 > 350 pmol/L; 20—dementia and hypertension; 21—transient ischaemic attack and serum vitamin B12 > 350 pmol/L; 22—transient ischaemic attack and dementia.

To examine which parameters could be clinically useful for assessing the risk of poststroke HF, we evaluated the predictive properties of all variables independently associated with poststroke HF as well as of SPTH (a significant independent determinant when subjects with history of stroke are compared with patients without CVD) and living in a permanent RCF (although not an independent but a significant and practically important indicator of poststroke HF). The relative risk (RR) estimates of characteristics associated with poststroke HF are depicted in Figure 1. Among elderly HF patients, risk of having a poststroke HF is greater 3.7 times in subjects with history of TIA and in permanent RCF residents, 3.2 times higher in females, 2.9 times in patients with dementia or hypertension, 2.2 times in those with SHPT, 2.1 times in subjects with higher serum vitamin B12 (>350 pmol/L), 2 times in patients with CAD, and 1.8 times in walking aid users. The sensitivity of these variables ranges between 83% (female sex) and 16% (TIA history, RCF resident) and the specificity between 90.5% (TIA history) and 30.0% (female sex), positive predictive value between 17% (female sex)—45.2% (TIA history, SHPT, and RCF resident) and 60% (vitamin B12 > 350 pmol/L), and negative predictive value between 79.1% (SHPT) and >90% (female sex, hypertension, and vitamin B12 > 350 pmol/L). 

The probability of poststroke HF increases, understandably, when two or more factors coexist. Measurement of serum PTH and vitamin B12 may provide additional prognostic information when results are combined with selected clinical factors. The highest RR demonstrates the following combinations: dementia and TIA (RR = 5.9), TIA and higher vitamin B12 level (RR = 5.4), dementia and hypertension (RR = 5.1), dementia and higher vitamin B12 level (RR = 4.4), and TIA and SHPT (RR = 3.1). The specificity of each of the pairs of variables shown in Figure 1 is 93% or higher, and the negative predictive value is >88.0%, while the sensitivity ranges only between 13.3% and 39.5%, and the positive predictive value ranges between 40.0% and 71.4%. 

### 3.6. Short-Term Outcomes in Poststroke Hip Fracture Patients

Data summarised in Table 6 shows that, in HF patients, history of stroke was associated with 2.7 higher incidence of postoperative myocardial injury defined as cTnI rise and being discharge to a long-term RCF but did not influence significantly the length of hospital stay (LOS) and the in-hospital mortality (5.0% versus 4.8% in subjects with and without history of stroke, resp.).

In the total cohort, SHPT was an independent predictor of poor outcomes. Among the poststroke HF patients, SHPT was present in all subjects who died, in 65.8% with myocardial injury, in 71.7% with prolonged hospital stay (>20 days), and in 73.2% discharged to a long-term RCF (all *P* < 0.01). Higher vitamin B12 levels (>350 pmol/L) in the total cohort were also associated with in-hospital death (OR = 7.33, 95%CI 1.57–34.14, *P* = 0.011) and prolonged LOS (OR = 1.98, 95%CI 1.16–3.37, *P* = 0.012). Higher vitamin B12 levels had all poststroke HF patients who died and 71.4% with prolonged LOS (*P* = 0.027).

### 3.7. Prefracture Use of Antiosteoporotic Therapy by Poststroke Hip Fracture Patients

Compared to the rest of the cohort in the poststroke HF group, the prefracture use of a bisphosphonate (15.0% versus 8.5%) and supplementation with vitamin D (15.0% versus 9.3%) and calcium (21.0% versus 11.0%) were slightly but not significantly higher (all *P* > 0.1) and far from adequate.

## 4. Discussion

### 4.1. Main Findings

This cross-sectional analysis of data from an observational, prospective study demonstrates a high prevalence of poststroke in elderly subjects among HF patients (1), describes the differences in the clinical profile, mineral-bone metabolism characteristics, and short-term outcomes in poststroke compared to nonstroke HF patients (2), emphasises the prefracture underuse of antiosteoporotic therapy (3), and presents prognostic indicators of having a poststroke HF (4). While some of our findings corroborate previous work, others are novel and will be discussed in more detail.

### 4.2. Prevalence and Clinical Profile

We found that among 761 consecutive older patients with osteoporotic HF, the prevalence of poststroke HF was 13.1%; it occurred on average 2.4 years after the stroke and in 68% of patients on the side of hemiplegia. Our findings on stroke prevalence are consistent with most reported data showing that stroke account for 7.3% to 15.3% of all HFs [35, 41, 43, 45], but higher than 3.9% in some studies [55] and lower than 38.5% in 1997 in a Swedish study [4]. Such differences might be attributable to age (our cohort was approximately 11 years older than the Swedish study: 84.1 versus 73 years, resp.), incidence rates of stroke survivors, environmental factors, and activity levels. Our data are in agreement with observations that most HFs occurred ipsilateral to the side of hemiplegia within 2-3 years after the stroke [4, 28, 30, 35, 37]. In concordance with other studies, we did not find differences between the poststroke and nonstroke groups with regard to patient age [35, 37], type of HF [35], smoking history, alcohol consumption, diabetes, AF [27], Parkinson's disease, COPD, and renal impairment nor most (22 of 25) routine laboratory parameters. 

The poststroke HF patients demonstrated a number of clinical and laboratory characteristics different from those in the rest of the cohort. The poststroke group showed a female predominance (independent of age), higher proportion of subjects living in long-term RCF, using walking aids, with dementia, having a history of TIA, hypertension, CAD, prior myocardial infarction, and ASA score ≥ 3. Among 25 laboratory parameters, higher serum vitamin B12 levels, leukocyte count, and lower mean corpuscular volume (MCV) in poststroke subjects were the only variables differing the groups. Our results are consistent with most previous reports of up to a 4-fold female predominance among poststroke HF persons [4, 25, 27, 28, 37, 45, 56], despite the age-specific incidence rates of stroke in women being lower than in men [57]. Only one study found a prevalence of men among poststroke HF patients [35]. Living in institutions [4], dementia [4], and ASA score ≥ 3 [35] is known to be associated with poststroke HF. The associations of TIA, hypertension, and CAD, established risk factors for stroke, with osteoporotic fractures [58–65] have also been documented. Our observations extend findings noted by others by providing data on a population with poststroke HF.

An unexpected finding was higher mean vitamin B12 concentrations in the poststroke HF patients, while the MCV was lower, and all other erythrocyte-related parameters were similar in both groups, suggesting the significant contribution of multiple factors (blood loss, inflammation, and malnutrition) other than vitamin B12 on erythropoiesis and haemoglobin status. Serum vitamin B12 levels were positively associated with history of stroke in crude and multivariate adjusted models irrespective of whether vitamin B12 was treated as a continuous or categorical variable (>350 pmol/L). Although some previous studies suggested that vitamin B12 deficiency by causing increased homocysteinaemia is a risk factor for osteoporosis and fragility fractures [66–71], recurrent stroke, vascular dementia, and Alzheimer's disease, existing data are inconclusive [72–76]. A reduction in HF rates in elderly Japanese stroke patients with residual hemiplegia treated with mecobalamine and folate was reported [68]. However, in general, causal relationships of low levels of vitamin B12 with reduced bone quality [77–80] and developing dementia [81, 82] are currently not supported. In poststroke patients no favourable effects of treatment with vitamin B12 (plus folic acid and vitamin B6) on risk of recurrent stroke, carotid intima-media thickness, myocardial infarction, or death have been observed [83–86]. Moreover, elevated vitamin B12 serum levels were associated, especially in the elderly, with symptomatic venous thromboembolism following major orthopaedic surgery of the low limb [87], mortality [88–90], carcinomas, haematological malignancies, and parenteral nutrition [51–53]. Vitamin B12 therapy increased cardiovascular risk in patients with diabetic neuropathy [91], cancer outcomes, and all-cause mortality in patients with CAD [92]. To the best of our knowledge, ours is the first study to show an association between higher vitamin B12 serum levels and poststroke HF. Understanding the underlying mechanisms will be a future research area of great interest.

### 4.3. Mineral and Bone Metabolism

With respect to indices of mineral and bone metabolism, we found that vitamin D insufficiency and excessive bone resorption were highly prevalent in both groups, but the poststroke group showed significantly higher mean levels of serum PTH and urinary bone resorption markers, lower values for osteocalcin, osteocalcin/BAP ratio, and a more pronounced imbalance between bone resorption and formation with resorption significantly exceeding formation. 

These results are in line with reports showing (1) a high prevalence of hypovitaminosis D and SHPT in poststroke patients [15, 69, 93–97] and HF patients [98–100], (2) an association of vitamin D insufficiency and especially elevated serum PTH levels with cardiovascular disease [31, 101–111] including stroke [69, 93, 112], hypertension [94, 104, 113–124], CAD [125], and carotid artery intima-media thickness (IMT) [126, 127], and (3) a high prevalence of osteoporosis in stroke patients [128] and a negative balance between bone formation and resorption in poststroke HF subjects [15–17, 21, 46, 47, 93, 129, 130]. 

Other researchers, however, reported no association between vitamin D status and stroke risk [131, 132], between PTH and blood pressure/hypertension [116, 118, 133] and between PTH and IMT [134]. In some studies, poststroke patients demonstrated significant BMD loss, while serum PTH and vitamin D levels and bone turnover markers were within normal range [26].

Not surprisingly, altered vitamin D and PTH levels, two pleiotropic factors with multiple physiological functions in calcium homeostasis, morphogenesis, cell proliferation, differentiation, and apoptosis, affect both bone and vascular health [135, 136]. The role of PTH as an indicator of poststroke HF deserves additional comment to avoid misinterpretation. In this study of HF subjects, serum PTH levels were associated with an increased risk of poststroke HF in a continuous fashion after adjusting for age and sex. The mean PTH levels and prevalence of SHPT were significantly higher in poststroke patients compared to the rest of the cohort (despite similar mean 25(OH)D levels and even slightly lower prevalence of hypovitaminosis D), and elevated PTH levels independently predicted history of stroke when comparison was made with patients without CVD but not with all nonstroke patients among which a significant proportion had CVD. These findings are in agreement with previous studies reporting that higher serum PTH levels are associated with stroke, hypertension, CAD, AF, and congestive heart failure [31, 69, 101, 106, 107, 109, 110, 125, 137–140]. PTH receptors have been found on cardiomyocytes [141] and endothelial cells [142, 143]. Serum PTH levels were shown to be an independent risk factor for cardiovascular events, cardiovascular mortality, and all-cause mortality, even in individuals with PTH within the normal range [109, 137, 140, 144, 145]. Altogether these data support the hypothesis that elevated PTH concentration is an abnormality of mineral metabolism affecting both the skeleton and vascular system (particularly the function of medium- and large-sized arteries and the myocardium), and therefore the presence of even mild PTH elevation is a condition that should be taken into account for the prevention of both CVD and fracture risks. 

Although vitamin D deficiency is known as the main cause of SHPT, clinical practice shows that the vitamin D-PTH relationship is complex. In elderly patients with long-standing hemiplegic stroke, hypovitaminosis D occurs without hyperparathyroidism [15, 16, 93] possibly because of immobilization-induced hypercalcaemia which may inhibit the compensatory PTH elevation; no correlation between serum calcium and PTH concentrations was seen in these patients [97]. In contrast, we found in poststroke HF subjects higher mean PTH levels and a higher SHPT prevalence despite similar mean 25(OH)D concentration and slightly lower prevalence of hypovitaminosis D compared to the nonstroke group, as well as a significant correlation between serum calcium and PTH levels in both groups. Taken together, these data indicate that the group of poststroke patients is clinically and biologically heterogeneous and in addition to hypovitaminosis D other factors attributable to SHPT must be taken into consideration.

In both groups no significant correlation between markers of bone turnover, except urinary markers of bone resorption, was present suggesting an imbalance between resorption and formation. However, uncoupling of these two processes, as indicated by the BSI index, was more prominent in the poststroke patients. Consistent with previous studies [47], we found that excess bone resorption in poststroke HF patients was near universal. Increased sclerostin production by osteocytes in response to skeletal unloading/immobilization has been proposed as an important molecular mechanism for acceleration of bone remodelling, with bone resorption exceeding formation [46, 146]. Reports on association between CVD and bone turnover markers are inconsistent [147, 148]. Increased bone resorption (high DPD) has been shown to be an independent predictor of CVD, including stroke [31, 33], and decreased bone formation, measured by bone histometry, was independently associated with increased coronary calcification [149]. 

Compared to the rest of the cohort, in poststroke HF patients, the mean BAP serum levels did not differ, but the mean OC concentration and the ratio OC/BAP were significantly lower. These may suggest impaired osteoblastic maturation, as BAP is expressed in the early period of osteoblastic differentiation, whereas OC is expressed in the later period [150]. This abnormality was associated with higher vitamin B12 levels. Indeed, vitamin B12 positively correlated with BAP in both groups, but patients with higher vitamin B12 levels had significantly lower mean OC/BAP ratio compared to those with vitamin B12 < 350 pmol/L. The poststroke subjects with higher vitamin B12 concentrations had significantly higher BAP levels and the lowest OC/BAP ratio, indicating that vitamin B12 may influence maturation of osteoblasts, which in turn may contribute to bone fragility and fractures. In vitro, vitamin B12 in low concentrations dose dependently increased alkaline phosphatase (ALP) activity of osteoblastic cells [151], while vitamin B12 deficiency did not affect the onset of osteoblast differentiation, maturation, and matrix mineralization [152] and had no influence on ALP expression in human osteoblasts [153] but increased homocysteine induced osteoclastogenesis in a dose-dependent manner [152]. These data indicates that vitamin B12 deficiency (when causing hyperhomocysteinaemia) may increase osteoclast formation and bone resorption, while higher B12 levels may affect osteoblast maturation. Reduction in serum OC/BAP ratio is associated with vertebral fractures independent of BMD in diabetic men [54]. In our study, vitamin B12 levels correlated with PTH. It appears, therefore, that higher serum B12 levels among poststroke HF patients in addition to SHPT and excess bone resorption may in part mediate the link between stroke and HF. The multivariate logistic regression analysis showed that higher serum vitamin B12 level was associated with poststroke HF independently of parameters of mineral and bone metabolism indicating that bone remodelling status, including osteoblast dysfunction, is incorporated in the model; this suggests that vitamin B12 is an important regulator of bone cell function and higher serum vitamin B12 levels may be directly linked to bone fragility in poststroke HF.

The next interesting finding of the study relates to phosphate homeostasis. As in other studies [47], the mean serum concentrations of calcium (albumin corrected), phosphate, and magnesium were within normal ranges and similar in both groups. However, upon classification of patients into two groups, with and without history of stroke, Pearson correlation results differed significantly. In the group with poststroke HF, serum phosphate level, though within the normal range, significantly and positively correlated with serum calcium and bone formation marker osteocalcin, as well as with both urinary bone resorption markers (DPD/Cr and NTx/Cr) and negatively with BSI. In the nonstroke group phosphate correlated positively only with BAP and DPD/Cr. In poststroke subjects, even small increases in phosphatemia are associated with increased bone resorption and decreased BSI, despite higher serum osteocalcin and calcium concentrations, both of which are negatively associated with eGFR. Notably, the phosphate levels correlated positively with in-hospital death. Even small increments in serum phosphate have been found to be associated with arterial calcification [154], adverse cardiovascular, and renal events and mortality [107, 155–158]. In the light of the above, our observation that, in the poststroke HF subjects, higher phosphate levels (without hyperphosphatemia) are associated with BSI, bone resorption markers, and osteocalcin, which also exerts a profound effect on energy metabolism [159, 160], can be interpreted as an additional factor contributing to the link between vascular dysfunction/calcification, subsequent stroke, and HF. However, whether higher phosphate levels (within the normal range) are directly implicated in the pathogenesis of poststroke HF or merely reflect mineral and bone metabolism status warrants further clarification.

Among several specific biological mechanisms that may underlie these associations of particular interest are fibroblast growth factor 23 (FGF-23) and Klotho proteins. FGF-23, an osteocytes-derived circulating hormone, that controls serum levels of phosphate, 1,25-dihydroxy vitamin D3, and PTH, interacts with RAAS [161] and may, therefore, affect both the cardiovascular system and skeleton. Elevated FGF-23 levels contribute to decreased 1,25-dihydroxy vitamin D3 levels [162], secondary hyperparathyroidism [163], suppression of osteoblast differentiation, and matrix mineralization in vitro and in vivo [122] and are associated with vascular calcification [157, 164], endothelial dysfunction [165, 166], cardiovascular disease [167], mortality [163, 167, 168], HF, and nonvertebral fractures in elderly men [169]. However, in some studies [170, 171] the relationship between FGF-23 and vascular calcification was not observed. 

Our results further support the link between mineral-bone metabolism abnormalities and stroke (and other CVD) and demonstrate that some metabolic interactions, particularly between phosphate and bone resorption markers, between vitamin B12 and OC/BAP ratio, are prevalent in poststroke HF subjects. Circulating 25(OH)D, PTH, phosphate, and vitamin B12 levels are linked to both stroke and HF, further accentuating their important underlying role in vascular and bone biology. 

Our final multivariate regression analysis demonstrated that six clinical characteristics (female sex, history of TIA, dementia, hypertension, CAD, and use of a walking aid) and higher serum B12 level are independent indicators/predictors for the development of HF. Because hypovitaminosis D was near universal among the HF patients, regardless history of stroke, and it is only one of the determinants of SHPT, it did not appear as an independent indicator of poststroke HF in our model.

### 4.4. Clinical Applications: Outcomes, Prediction, and Prevention Therapies

This study demonstrates that compared to the rest of the cohort, poststroke HF patients had approximately a 3-fold higher incidence of postoperative myocardial injury (as evidenced by cardiac troponin I rise) and need to be discharge to a long-term RCF. Our observations regarding the discharge status are consistent with some studies [4], but not others [35]. Similarly, in agreement with some [35] but not all [4, 172] previous reports, we found that in-hospital mortality [35] and LOS did not differ significantly between patients with and without history of stroke. Of note, SHPT and higher vitamin b12 levels were associated with in-hospital death and duration of hospital stay.

Approximately one in 7 older stroke survivors will suffer a HF which is associated with serious short- and long-term health consequences. To design preventive strategies, it is important to identify which persons are at the greatest risk. Our data suggests that compared with nonstroke HF patients, the relative risk of poststroke HF is 1.8–3.7 times higher in females, subjects with history of TIA, dementia, hypertension, CAD, using a walking aid, living in a permanent RCF, and having SHPT or higher vitamin B12 levels (>350 pmol/L). Presence of any two of these characteristics increases the RR to 2.5–5.9. SHPT and higher vitamin B12 levels are promising biomarkers for identification of older adults at increased risk of poststroke HF, and their measurement provides additional prognostic information when combined with clinical factors. These findings suggest that the combined effect of having a stroke, being a female and one of the above mentioned conditions, might significantly contribute to and predict an HF.

It should, however, be pointed out that, in general, the specificity of analysed factors was higher than the sensitivity, and the negative predictive value (usually above 88%) was higher than the positive predictive value with exception for female sex and hypertension which have sensitivity of 83% and 76.3%, respectively. That means that these two factors identify more poststroke persons at risk for HF, while the probability that the complication will not occur is low only when any of the studied characteristics is not present. Antiosteoporotic therapy for stroke survivors has not been adopted into current guidelines and routine clinical practice possibly because accurate predictive indicators were not available. Although the prognostic tools for poststroke HF need further improvement, the above-listed factors may be helpful in evaluating the risk of developing HF and deciding who is most likely to benefit from intervention. 

Only a small proportion of our poststroke patients (15%) received antiosteoporotic medications prior to their HF. In concordance with our observations, use of bisphosphonates was low (2.1%–2.7%) and did not differ between poststroke and nonstroke groups in other countries [30, 36]. Many HF are preventable, and the effectiveness of antiresorptive agents and vitamin D in fracture risk reduction in stroke survivors has been demonstrated [24, 96, 97, 173–175], but the gap between existing evidence-based and actual care remains huge. The results of this study in keeping with previous reports [56, 176] emphasise the importance of assessment for and prevention of poststroke osteoporosis and falls. Currently recommended measures in addition to bisphosphonates, vitamin D, and calcium supplementation include sunlight exposure [177], improving physical activity, exercises aimed at preventing falls [178–181], and hip protectors [182, 183]. In poststroke patients with immobilization-induced hypercalcaemia, calcitonin (in combination with vitamin D) may be helpful [97, 184]. Antiosteoporosis medications are generally safe and well tolerated, but the extraskeletal risks of bisphosphonates and calcium supplements, although rare, should also be considered [185–187]. To date there have been no studies on the use of bone anabolic agents (PTH 1–34, teriparatide), strontium ranelate, or denosumab in stroke survivors.

It should be emphasized that CVDs, especially history of TIA, hypertension, and CAD, are not only important and modifiable hazards to the brain but also independent indicators/predictors of poststoke HF. Therefore, correction of hypovitaminosis D and SHPT, which plays a specific mechanistic role in the pathogenesis of osteoporosis and CVD, and adequate antiosteoporotic therapy in addition to antihypertensive, antithrombotic, and cholesterol lowering treatment may markedly contribute to both stroke and fracture prevention. Our data supports reports challenging the existing approach to homocysteine lowering with vitamin B therapy and suggests a need to consider the association of higher serum vitamin B12 levels and adverse effects in stroke patients. Although the serum B12 level below which total homocysteine and methylmalonic acid levels become elevated is 400 pmol/L [188], we found that the serum vitamin B12 concentration > 350 pmol/L is associated with adverse clinical outcomes. Vitamin B therapy may thus be beneficial in persons with B12 deficiency but may be harmful when the serum B12 concentration is higher (even within the reference range), especially in subjects with impaired renal function.

Over recent decades, stroke mortality was falling, and the population of stroke survivors was steadily increasing. To reduce the public health burden of the substantial proportion of poststroke subjects with HF secondary prevention programs directed on progression of osteoporosis should be included in the guidelines for management of these patients.

## 5. Limitations

Our study has several limitations. First, owing to the cross-sectional study design, causal effects cannot be determined. Second, in this single-centre study there were only 100 poststroke HF patients; therefore, its statistical power might be insufficient for detecting all relationships. Third, serum levels of ionized calcium, 1,25(OH)2D, FGF-23, and Klotho were not examined, and the dietary phosphorus and calcium intake were not assessed, although these variables contribute to mineral-bone metabolism and can provide additional insights to underlying mechanisms. Fourth, the cohort was comprised predominantly of Caucasians, and the findings may not be applicable to other racial groups. The strengths of this study include a comprehensive assessment of a relatively large well-characterized cohort of consecutive older patients with osteoporotic HF representative of real-life practice and consideration of multiple potential biologic factors, some of which have not been studied extensively previously. 

## 6. Conclusions

In the elderly, approximately one in seven HF occurs in stroke survivors, usually within the first 2.4 years after the stroke. Poststroke HFs are prevalent in women and associated with dementia, history of TIA, hypertension, CAD, hypovitaminosis D, SHPT, high bone resorption, higher serum vitamin B12 levels, and poorer outcomes (postoperative myocardial injury and RCF need). In stroke survivors prefracture antiosteoporotic therapy is rarely used; early implementation of fracture prevention strategies is urgently needed.

## Figures and Tables

**Table 1:** Sociodemographic and clinical characteristics in older hip fracture patients with and without history of stroke.

Characteristics	HF poststroke (*n* = 100)	HF without history of stroke (*n* = 661)	*P* value
Age, years (mean ± SD)	84.1 ± 7.72	81.9 ± 7.96	0.120
Age, females, years (mean ± SD)	84.1 ± 8.03	82.5 ± 7.59	0.264
Age, males, years (mean ± SD)	83.3 ± 4.92	80.4 ± 8.62	0.521
Females, %	83.0	70.0	**0.011**
Admitted from long-term RCF, %	50.0	26.9	**0.004**
Cervical/trochanteric HF, *n*	55/45	346/315	0.698
Coronary artery disease, %	35.0	20.9	**0.002**
Previous myocardial infarction, %	10.0	4.6	**0.024**
Hypertension, %	60.0	42.1	**0.001**
Atrial fibrillation, %	18.0	12.5	0.092
TIA, %	16.0	6.1	**0.001**
Dementia, %	47.0	26.4	**0.001**
Diabetes mellitus, %	19.0	16.8	0.479
COPD, %	13.0	11.1	0.583
Parkinson's disease, %	3.0	4.6	0.473
eGFR < 60 mL/min/1.73 m², %	47.0	43.4	0.650
eGFR < 30 mL/min/1.73 m², %	7.9	4.5	0.371
ASA score ≥3, %	96.9	68.2	**0.001**
Current Smoker, %	6.0	5.3	0.785
Ex-smoker, %	12.0	9.1	0.365
*Alcohol overuser, %	5.0	5.6	0.795
User of walking device, %	50.0	32.2	**0.001**

HF: hip fracture; RCF: residential care facility; TIA: transient ischaemic attack; eGFR: estimated glomerular filtration rate; ASA: American Society of Anaesthesiologists; COPD: chronic obstructive pulmonary disease. *Three or more times per week.

**Table 2:** Haematologic, renal, liver, and thyroid parameters in older hip fracture patients with and without history of stroke.

Characteristics	HF poststroke	HF without history of stroke	*P* value
Erythrocyte count, ×10^12^/L	4.21 ± 0.61	4.08 ± 0.61	0.217
Haemoglobin, g/L	126.0 ± 18.9	124.7 ± 16.9	0.601
Leukocyte count, ×10^9^/L	12.0 ± 4.22	10.5 ± 4.24	**0.034**
Lymphocyte count, ×10^9^/L	1.24 ± 1.39	1.31 ± 1.22	0.742
MCV, fl	87.4 ± 16.5	90.8 ± 5.98	**0.019**
MCH, pg/cell	30.3 ± 3.35	30.7 ± 2.29	0.290
MCHC, g/L	338.0 ± 19.4	340.7 ± 37.1	0.660
RDW, %	15.1 ± 2.05	15.2 ± 9.53	0.942
Iron, *μ*mol/L	4.81 ± 4.12	5.33 ± 4.39	0.507
Transferrin, g/L	1.68 ± 0.49	1.72 ± 0.48	0.582
Transferrin saturation, %	11.08 ± 10.07	11.65 ± 8.36	0.716
Ferritin, mg/L	313.1 ± 244.9	298.9 ± 283.3	0.778
Vitamin B12, pmol/L	482.6 ± 321.0	376.0 ± 256.0	**0.027**
Folic acid (serum), nmol/L	25.2 ± 16.7	26.1 ± 15.4	0.747
Urea, mmol/L	7.9 ± 4.30	7.8 ± 3.67	08.37
Creatinine, mmol/L	92.0 ± 43.86	93.6 ± 57.24	0.873
eGFR, mL/min/1.73 m^2^	62.5 ± 25.2	64.9 ± 23.2	0.561
Albumin, g/L	36.6 ± 6.0	37.0 ± 6.4	0.723
Bilirubin, *μ*mol/L	13.0 ± 5.95	12.2 ± 7.62	0.503
ALT, U/L	20.4 ± 12.8	21.4 ± 22.3	0.801
ALP, U/L	103.8 ± 44.3	105.9 ± 85.8	0.882
GGT, U/L	55.8 ± 90.5	54.3 ± 98.6	0.928
TSH, *µ*U/L	1.25 ± 1.47	1.60 ± 2.31	0.361
T_4_, pmol/L	16.02 ± 3.61	15.98 ± 3.54	0.949
CRP, mg/L	137.1 ± 76.7	129.2 ± 83.1	0.583

The values are expressed as mean ± standard deviation (SD). HF: hip fracture; MCV: mean corpuscular volume; MCH: mean corpuscular haemoglobin; MCHC: mean corpuscular haemoglobin concentration; RDW: red-cell distribution width; eGFR: estimated glomerular filtration rate; ALT: alanine aminotransferase; ALP: alkaline phosphatase; GGT: gamma glutamyltransferase; TSH: thyroid-stimulating hormone; T_4_: total thyroxin.

**Table 3:** Parameters of mineral and bone metabolism in older hip fracture patients with and without history of stroke.

Parameter	Poststroke HF	Without stroke HF	*P* value
Serum calcium*, mmol/L	2.27 ± 0.12	2.28 ± 0.13	0.730
Serum phosphate, mmol/L	0.89 ± 0.30	0.96 ± 0.50	0.428
Serum magnesium, mmol/L	0.77 ± 0.12	0.78 ± 0.13	0.887
25(OH)vitamin D, nmol/L	40.1 ± 20.05	36.8 ± 17.97	0.319
PTH, pmol/L	9.0 ± 6.66	7.6 ± 5.41	**0.019**
Osteocalcin, ng/L	13.8 ± 8.44	18.0 ± 16.18	**0.025**
BAP, IU/L	25.5 ± 9.76	26.8 ± 14.97	0.624
Osteocalcin/BAP ratio	0.59 ± 0.37	0.78 ± 0.78	**0.026**
Urinary DPD/Cr, nmol/*μ*mol	16.0 ± 14.02	12.0 ± 5.53	**0.004**
Urinary NTx/Cr, nmol/*μ*mol	207.9 ± 279.0	149.7 ± 141.3	0.056
BSI	−12.5 ± 14.8	−8.6 ± 6.1	**0.009**
PTH > 6.8 pmol/L, %	50.0	34.2	**0.046**
25(OH)D < 50 nmoL/L, %	68.0	81.7	**0.044**
25(OH) vitamin D < 25 nmol/L, %	27.0	31.0	0.626
Osteocalcin < 14.0 ng/L, %	57.0	51.9	0.582
BAP < 14.0 IU/L, %	11.0	9.3	0.734
Urinary DPD/Cr > 7.5 nmol/*μ*mol,%	90.0	81.9	0.189
Urinary NTx/Cr > 65 nmol/*μ*mol, %	78.0	71.6	0.444

The values are expressed as mean ± standard deviation (SD) or percentage.

*Adjusted for serum albumin. PTH: parathyroid hormone; BAP: bone specific alkaline phosphate; DPD/Cr: deoxypyridinoline corrected for urinary creatinine concentration; NTx/Cr: cross-linked N-telopeptide of type 1 collagen corrected for urinary creatinine concentration; BSI: bone balance status index.

**Table 4:** Pearson correlation coefficients among selected parameters of mineral and bone metabolism and their relation to age and renal function in older hip fracture (HF) patients with and without history of prefracture stroke.

	Poststroke HF		HF without history of stroke	
	*r*	*P*	*r*	*P*
Calcium-PTH	−0.328	0.047*	−0.273	<0.001
Calcium-osteocalcin	0.3226	0.052*	0.139	0.034*
Calcium-BAP	0.137	0.434	0.16	0.015
Osteocalcin-eGFR	−0.525	0.001	−0.346	<0.001
Calcium-eGFR	−0.464	0.004	−0.075	0.250
DPD/Cr-NTx/Cr	0.779	<0.001	0.380	<0.001
DPD/Cr-BSI	−0.926	<0.001	−0.690	<0.001
NTx/Cr-BSI	−0.952	<0.001	−0.876	<0.001
Age-eGFR	−0.443	0.005	−0.337	<0.001
Phosphate-calcium	0.415	0.011	0.055	0.399
Phosphate-DPD/Cr	0.580	0.001	0.208	0.003
Phosphate-osteocalcin	0.489	0.002	0.123	0.061
Phosphate-BAP	0.215	0.216	0.169	0.01
Phosphate-NTx/Cr	0.478	0.006	0.079	0.263
Phosphate-BSI	−0.531	0.003	−0.109	0.126
Phosphate-eGFR	−0.32	0.054*	−0.173	0.008
PTH-vitamin B12	0.365	0.028*	0.016	0.817
BAP-vitamin B12	0.442	0.009	0.188	0.006

Values for all variables other than age were log transformed before analysis.

PTH: parathyroid hormone; eGFR: estimated glomerular filtration rate; DPD/Cr: urinary deoxypyridinoline corrected for urinary creatinine concentration; NTx/Cr: cross-linked N-telopeptide of type 1 collagen corrected for urinary creatinine concentration; BSI: bone balance status index. *Nonsignificant (*P* > 0.005) correlations when Bonferroni and Sidak adjustments for multiplicity were adopted.

**Table 5:** Clinical and laboratory characteristics independently associated with poststroke hip fracture.

Characteristic	Model 1			Model 2			Model 3		
	OR	95% CI	*P* value	OR	95% CI	*P* value	OR	95% CI	*P* value
Female sex	3.73	1.11–12.59	0.034	3.64	1.17–11.42	0.026	3.58	1.03–12.45	0.045
TIA	5.17	1.62–16.48	0.005				5.17	1.59–16.79	0.006
Dementia	4.16	1.56–11.05	0.004				4.14	1.79–9.59	0.001
Hypertension	3.34	1.37–8.14	0.008				3.20	1.29–7.91	0.012
CAD	2.70	1.03–7.07	0.043				2.41	0.97–5.97	0.057
Use of walking aid	2.20	1.03–5.09	0.043				2.47	1.08–5.65	0.032
Vitamin B12 > 350 pmol/L				2.35	1.05–5.49	0.039	2.33	1.03– 5.25	0.042
*R* ^2^	0.221			0.126			0.225		
Mean V1F	1.19			1.25			1.03		

OR: odds ratio; CI: confidence interval; TIA: transient ischaemic attack; CAD: coronary artery disease; VIF: variance inflation factor.

Model 1 (clinical parameters) adjusts for age, sex, dementia, hypertension, CAD, history of myocardial infarction, TIA: atrial fibrillation, renal impairment (eGFR < 60 mL/min/1.73 m^2^), use of walking aid, and living in a long-term residential care facility.

Model 2 (laboratory parameters) adjusts for age, sex, vitamin D insufficiency (25(OH)D < 50 nmol/L), elevated PTH (>6.8 pmol/L), and high bone resorption markers (DPD/Cr > 7.5 mmol/*μ*mol, NTX/Cr > 65 mmol/*μ*mol).

Model 3 (combined clinical and laboratory parameters) adjusts for al variables in Models 1 and 2.

**Table 6:** Effect of history of stroke on short-term outcomes in older hip fracture patients.

Outcome	OR	95% CI	*P* value
Postoperative myocardial injury*	2.68	1.31–5.47	**0.007**
LOS ≤ 10 days	0.92	0.44–1.90	0.816
LOS ≥ 20 days	1.35	0.66–2.77	0.413
New discharges to long-term RCF	2.69	1.11–6.54	**0.028**
In-hospital death	1.07	0.23–5.00	0.927

Adjusted for age and sex.

*Cardiac troponin I rise (>0.06 *μ*g/L). LOS: length of hospital stay.
